# Isolation of Bioactive Compounds That Relate to the Anti-Platelet Activity of *Cymbopogon ambiguus*

**DOI:** 10.1093/ecam/nep213

**Published:** 2011-02-14

**Authors:** I. Darren Grice, Kelly L. Rogers, Lyn R. Griffiths

**Affiliations:** ^1^Institute for Glycomics, Gold Coast campus, Griffith University, Queensland, 4222, Australia; ^2^Plate-forme d'imagerie dynamique, Institut Pasteur, Paris Cedex 15, France; ^3^Genomics Research Centre, Gold Coast campus, Griffith University, Queensland, 4222, Australia

## Abstract

Infusions and decoctions of *Cymbopogon ambiguus* have been used traditionally in Australia for the treatment of headache, chest infections and muscle cramps. The aim of the present study was to screen and identify bioactive compounds from *C. ambiguus* that could explain this plant's anti-headache activity. A dichloromethane extract of *C. ambiguus* was identified as having activity in adenosine-diphosphate-induced human platelet aggregation and serotonin-release inhibition bioassays. Subsequent fractionation of this extract led to the isolation of four phenylpropenoids, eugenol, elemicin, eugenol methylether and *trans*-isoelemicin. While both eugenol and elemicin exhibited dose-dependent inhibition of ADP-induced human platelet serotonin release, only eugenol displayed potent inhibitory activity with an IC_50_ value of 46.6 **μ**M, in comparison to aspirin, with an IC_50_ value of 46.1 **μ**M. These findings provide evidence to support the therapeutic efficacy of *C. ambiguus* in the non-conventional treatment of headache and inflammatory conditions.

## 1. Introduction

The native Australian lemongrass species, *Cymbopogon ambiguus* A. Camus. (Poaceae) is a strongly aromatic perennial grass found on rocky hillsides throughout the Northern Territory of Australia [1]. The leaves have been used traditionally to treat chest infections, sores, muscle cramps as well as headache and associated complaints (infusions and decoctions) [1–3]. Interestingly, a study on Australian medicinal plants identified very weak anti-viral (Ross River Virus) activity in a leaf extract of *C. ambiguus* [4], while another study found no anti-bacterial activity in extracts of Australian *C. ambiguus* against four Gram-positive and four Gram-negative bacterial species [5]. Little is known about the chemical constituents present in *C. ambiguus* apart from a GC-MS study by Barr et al. [3], which identified camphene, borneol, limonene, *α*-pinene, *α*-terpineol, camphor, isoborneol, 4-terpineol, myrcene, *β*-ocimene as being present in the essential oil. Nothing has been reported to date in relation to substantiating its use as a non-conventional traditional remedy for headache, apart from our original report showing that dichloromethane (DCM) (more potent extract) and methanolic (MeOH) extracts displayed potent inhibition of human platelet aggregation and serotonin (5-HT, 5-hydroxytryptamine) release [6]. To further investigate the basis for use of *C. ambiguus* as a remedy for headache, we report here the isolation and identification of principal bioactive constituents causing inhibition of human platelet aggregation and serotonin (5-HT) release.

*Cymbopogon ambiguus* is a headache remedy used in Australian traditional medicine for generalized headache disorders. Headaches associated with nausea, such as migraine are complex disorders, with abnormalities in platelet function reported. Altered 5-HT transport, decreased platelet 5-HT content, altered platelet cytosolic free-calcium concentrations and varying sensitivity to platelet agonists such as adenosine diphosphate (ADP) and collagen have been reported in association with headache/migraine episodes [7–14]. The serotonergic system is thus thought to play an important role in the pathophysiology of these disorders [15–20]. We and others have previously reported on the use of platelets as a model to assess the therapeutic serotonergic potential of tested chemicals in relation to potential headache/migraine treatment [6, 21, 22].

*Cymbopogon ambiguus* has been used as a traditional Australian aboriginal headache treatment and our studies have shown that it displays potent anti-platelet activity. To identify the bioactive constituent(s) responsible for this activity, lemongrass leaves were sequentially extracted with DCM then MeOH. Fractionation of the DCM extract led to the identification of four phenylpropenoids as the principal constituents. We describe herein the isolation and purification of these four phenylpropenoids from *C. ambiguus* along with pharmacological evaluation of two of these compounds. This is the first report identifying specific constituents that underpin the use of *C. ambiguus* as a non-conventional traditional Australian anti-headache medicine.

## 2. Methods

### 2.1. Plant Collection

*Cymbopogon ambiguus* (whole plant) was collected in 1998 in the vicinity of Alice Springs, Northern Territory, Australia. Identity of the material was confirmed by the Alice Springs Herbarium, with voucher specimens deposited at the Herbarium and also in the Genomics Research Centre, Gold Coast campus, Griffith University (sample ID: DN3081).

### 2.2. Extraction and Isolation

Dried whole plants (283 g) of *C. ambiguus* were powdered and exhaustively extracted (vigorous stirring in 2.5 l of solvent for 3 h at 22°C, then procedure repeated) with DCM followed by MeOH at room temperature The DCM extract was then concentrated and dried under reduced pressure (*∼*50 mmHg, 30°C) to give a dark green aromatic solid residue (7.53 g) and the residue loaded onto a normal phase chromatography column (silica gel, 150 g, Merck, 60 *μ*M, 4.3 × 11.7 cm). The column was then eluted by passing 600 ml volume through the column from 10% hexane in chloroform, to 100% hexane in 10% increments. Fractions eluted with 50% and 60% hexane were both found to exhibit significant inhibition of ADP-induced platelet [^14^C]5-HT release. Analytical HPLC [Luna C_18_, 5 *μ*M analytical, 4.6 mm × 250 mm, photo-diode-array (PDA) detection at 270 nm, 1.0 ml min^−1^, method: 25%–35% acetonitrile/H_2_O gradient over 10 min, followed by an increase to 45% acetonitrile over a further 50 min] of these fractions indicated five common constituents, which were separated by semi-preparative HPLC [Luna C18, 5 *μ*M, 10 mm × 250 mm, PDA, 4.5 ml min^−1^, method as above] to give the known compounds: eugenol (4-allyl-2-methoxyphenol) (**1**) (75.4 mg, 0.02% w/w); elemicin (5-allyl-1,2,3-trimethoxybenzene) (**2**) (520 mg, 0.18% w/w); eugenol methylether (4-allyl-1,2-dimethoxybenzene) (**3**) (322 mg, 0.11% w/w); and *trans* iso-elemicin (**4**) (1,2,3-trimethoxy-5-(1-propenyl) benzene) (162 mg, 0.06% w/w) (Figure 3).

### 2.3. Chemicals and Solvents

ADP, acetyl salicylic acid (ASA or aspirin), dimethyl sulfoxide (DMSO) and deuterated chloroform (CDCl_3_) were all obtained from Sigma chemical company (St Louis, USA). ^14^C-5-hydroxytryptamine (^14^C-5-HT) (Amersham, specific activity 57 mCi mmol^−1^, 50 *μ*Ci ml^−1^). All solvents used were of HPLC grade, including acetonitrile (CH_3_CN) (omnisolv. EM Science, Merck), chloroform (CHCl_3_) (chromasolv. Riedel-de Haen), DCM (chromosolv. Riedel-de Haen), ethyl acetate (Banksia Scientific Co. Pty. Ltd.), hexane (Mallinckrodt chromAR HPLC) and methanol (Omnisolv, EM Science, Merck).

### 2.4. Characterization of Isolated Compounds

Structural characterization of the four phenylpropenoids was achieved by mass spectrometry (MS) (Bruker Daltonics BioAPEX 47e), extensive NMR experiments (Bruker AC-300, Karlsruhe, Germany) (^1^H, ^1^H-^1^H COSY, ^1^H-^13^C HMBC, JMOD) and comparison of ^1^H NMR (300 MHz, CDCl_3_) data (see below) with previously reported assignments. Eugenol (**1**) [23]: ^1^H NMR *δ* 3.33 (d, 2H, ^3^ *J* = 6.7 Hz, C**H_2_**); 3.88 (s, 3H, OC**H_3_**); 5.06 (dd, 1H, ^2^ *J*  =  2 Hz, ^3^ *J*_cis_ = 10 Hz, HC**H**CHCH_2_); 5.07 (dd, 1H, ^2^ *J*  =  2 Hz, ^3^ *J*_trans_ = 17 Hz, **H**CHCHCH_2_); 5.50 (O**H**); 5.93 (ddd, 1H, ^3^ *J*_cis_  = 10 Hz, ^3^ *J*_trans_ = 17 Hz, ^3^ *J* = 6.7 Hz, HCHC**H**CH_2_); 6.67–6.72 (m, 2H, CCHC**H**C(OH), CC**H**C(OCH_3_)); 6.86 (dd, 1H, *J*_ortho_ = 8.5 Hz, *J*_meta_ = 4.2 Hz, CC**H**CHC(OH). MS calculated for C_10_H_13_O_2_  [*M *+ H]^+^: 165.08. Found: 165.08. Elemicin (**2**), [24]: ^1^H NMR *δ* 3.33 (d, 2H, ^3^ *J* = 6.6 Hz, C**H_2_**); 3.82 (s, 3H, OC**H_3_**); 3.84 (s, 6H, (**H**_3_CO)CC(OC**H**_3_)C(OC**H**_3_); 5.08 (dd, 1H, ^2^ *J* = 1.8 Hz, ^3^ *J*_cis_ = 10 Hz, HC**H**CHCH_2_); 5.11 (dd, 1H, ^2^ *J* = 1.8 Hz, ^3^ *J*_trans_ = 17 Hz, **H**CHCHCH_2_); 5.95 (ddd, 1H, ^3^ *J*_cis_ = 10 Hz, ^3^ *J*_trans_ = 17 Hz, ^3^ *J* = 6.6 Hz, HCHC**H**CH_2_); 6.40 (s, 2H, 2 × aromatic **H**). MS calculated for C_12_H_16_O_3_Na [*M* + Na]^+^: 231.11. Found: 231.11. Eugenol methylether (**3**) [23]; ^1^H NMR *δ* 3.31 (d, 2H, ^3^ *J* = 6.6 Hz, HC = C**H_2_**); 3.84 (s, 3H, OC**H_3_**); 3.85 (s, 3H, OC**H_3_**); 5.04 (dd, 1H, ^2^ *J* = 1.5 Hz, ^3^ *J*_cis_ = 10.1 Hz, HC**H**CHCH_2_); 5.05 (dd, 1H, ^2^ *J* = 1.5 Hz, ^3^ *J*_trans_ = 16.8 Hz, **H**CHCHCH_2_); 5.95 (ddd, 1H, ^3^ *J*_cis_ = 10.1 Hz, ^3^ *J*_trans_ = 16.8 Hz, ^3^ *J* = 6.6 Hz, HCHC**H**CH_2_); 6.75 (m, 3H, ^3^ *J*_ortho_ = 8.4 Hz, ^4^ *J*_meta_ = 1.4 Hz, 3 × aromatic **H**). MS calculated for C_11_H_15_O_2 _[*M *+ H]^+^: 179.09. Found: 179.09. *trans*-Isoelemicin (**4**) [25]; ^1^H NMR *δ* 1.86 (dd, 3H, ^3^ *J* = 6.4 Hz, ^4^ *J* = 1.5 HCC**H_3_**); 3.81 (s, 3H, OC**H_3_**); 3.83 (s, 6H, 2 × OC**H_3_**); 6.13 (dq, 1H, ^3^ *J*_trans_ = 15.6 Hz, ^3^ *J* = 6.4 Hz, HC**H**CHCH_2_); 6.31 (dd, 1H, ^4^ *J*  =  1.5 Hz, ^3^ *J*_trans_ = 15.6 Hz, **H**CCHCH_3_); 6.54 (s, 2H, 2 × aromatic **H**). MS calculated for C_12_H_17_O_3 _[*M *+ H]^+^: 209.11. Found: 209.11.

### 2.5. Platelet Aggregation and [^14^C]-5HT Release Bioassays

The bioassays utilised are based on a modified published method, described previously by our group [26] and by Groenewegen and Heptinstall [27]. Fresh blood (45 ml) was collected from healthy human volunteers (20- to 35-year old, not taking aspirin or other drugs likely to interfere with platelet function for at least 2 weeks prior to blood sampling). Platelet aggregation and 5-HT release were measured simultaneously. [^14^C]5-HT (6 *μ*l) (specific activity 50 *μ*Ci ml^−1^) was added to 9 ml of citrated blood to label intracellular storage granules. Platelet-poor plasma (PPP) and platelet-rich plasma (PRP) fractions were prepared, with the PRP being adjusted to 300 × 10^9^ platelets/l by dilution with PPP. A sample consisting of 450 *μ*L of PPP and 100 *μ*L of either, PBS with 1% ethanol or DMSO (control), or the test sample, was initially measured in order to set 100% aggregation (a decrease in optical density reflects an increase in platelet aggregation). The final concentration of ethanol or DMSO did not exceed 0.2% (v/v). Labeled PRP (450 *μ*l) was then stirred (1000 r.p.m. at 37°C) for 3 min in the presence of 100 *μ*l of the *C. ambiguus* extract (or isolated compound) or PBS containing either 1% ethanol or DMSO (control). ADP (50 *μ*l) was then added to samples, and platelet aggregation was monitored for 6 minutes. Platelet aggregation was measured in a four-channel platelet aggregometer (Monitor IV plus, Helena Laboratories, Beaumont, USA). Samples of PRP were incubated without the addition of ADP to measure the amount of [^14^C] 5-HT that was not taken up by platelets, and which may have been spontaneously released during the procedure (blank). ASA (50 *μ*l, 14 mm) was then added to irreversibly inhibit further 5-HT release, and samples were placed on ice for *∼*5 min. Samples were then centrifuged (6000 r.p.m.) for 8 min, and duplicate 50 *μ*l aliquots were taken from the supernatant and counted (Wallac 1450, MicroBeta counter) for determination of % [^14^C] 5-HT release. Percentage 5-HT release was calculated as follows:

(1) Amount  of  [C14]5−HT−blankTotal  counts−blank×100%.

IC_50_ values were generated using the software of Graphpad prism (3.0).

### 2.6. Bioethics Clearance

Blood samples for the platelet studies were collected by a qualified phlebotomist and the study had full ethical clearance from the Griffith University Ethics Committee for experimentation on human subjects. Informed written consent was obtained from all participants.

### 2.7. Statistical Analysis

Platelet aggregation and 5-HT release response, was expressed as a percentage of the control value. IC_50_ values (concentration producing 50% inhibition of the maximum response) were calculated by non-linear regression analysis of the dose-response curves using the software of Graphpad Prism (3.0). Results are presented as the means ± SEM of *n* experiments.

## 3. Results

### 3.1. Extraction

Both DCM and MeOH extracts of *C. ambiguus* produced a dark green residue that was strongly aromatic (lemon scented). The total weight of the DCM extract after drying was 7.53 g, and for the MeOH extract, 16.84 g.

### 3.2. Isolation and Structural Characterization

**Figure 1:**
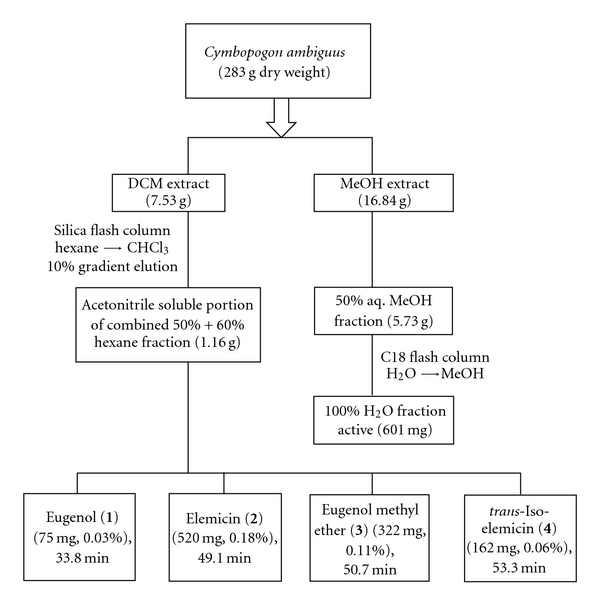
Bioassay-guided fractionation of *C. ambiguus* (whole plant) to isolate compounds (1–4).

**Figure 2:**
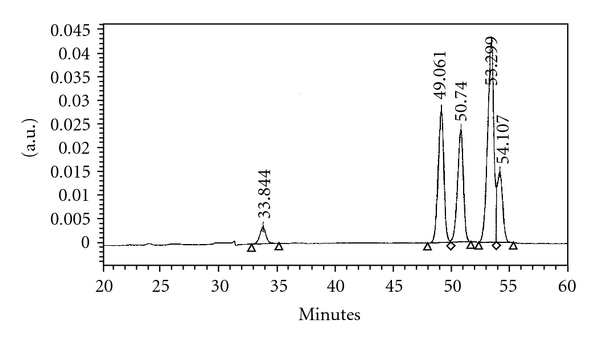
HPLC chromatogram of the combined 50 : 50 and 60 : 40 hexane/CHCl_3_ fractions: eugenol (33.8 min); elemicin (49.1 min); eugenol methylether (50.7 min); *trans*-iso-elemicin (53.3 min).

Silica column chromatography (10% gradient increments from 10 : 90 hexane : CHCl_3_ through to 100 : 0 hexane : CHCl_3_ elution) of the DCM fraction eluted an active 50 : 50–60 : 40 hexane fraction. The dried residue (weighing *∼*1.16 g) was resuspended in CH_3_CN and fractionation of the soluble components utilising HPLC, afforded five compounds (Figures 1 and 2). Four of these compounds were purified further (purity was ascertained by HPLC analysis) and then subjected to MS and NMR analysis. The fifth compound (eluting at 54.11 min) (Figure 2) was not successfully purified and was therefore not structurally characterised in these studies.

**Figure 3:**
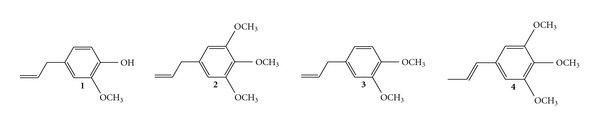
Structure of the four phenylpropenoides [eugenol (4-allyl-2-methoxyphenol) (**1**); elemicin (5-allyl-1,2,3-trimethoxybenzene) (**2**); eugenol methylether (4-allyl-1,2-dimethoxybenzene) (**3**) and *trans* iso-elemicin (1,2,3-trimethoxy-5-(1-propenyl) benzene) (**4**)] isolated from the Australian native lemongrass species *C. ambiguus*.

The assignment of the structures for eugenol, elemicin, eugenol methylether and *trans*-iso-elemicin (Figure 3), which are known compounds, was established on the basis of the ^1^H NMR and MS data. Chemical shifts were identical to those previously reported (see Methods section—Characterization of Isolated Compounds). The chemical structure and identity of each compound is shown in Figure 3. The isolation of these compounds was guided by the activity of the active hexane fraction (50% and 60%) in the ADP-induced platelet 5-HT release bioassay. Both eugenol and elemicin were investigated pharmacologically in further studies.

### 3.3. Platelet Aggregation and [^14^C]-5HT Release Bioassay

**Figure 4:**
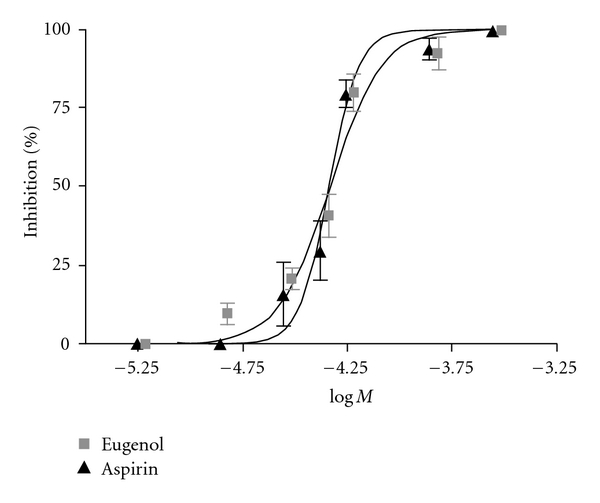
Dose-dependent inhibition of ADP (2 *μ*M)-induced human platelet [^14^C]5-HT release. Results represent the mean of duplicate analyses performed on PRP obtained from four different individuals.

For the platelet aggregation and 5-HT release studies, a submaximal concentration of ADP was used to stimulate the platelet response. Elemicin's affect on inhibiting ADP-induced platelet aggregation was less potent than eugenol in our results and therefore we focussed our interests on eugenol. Effects of increasing eugenol concentrations (0, 46, 61 and 152 *μ*M) on the ADP-induced platelet aggregation clearly showed that concentrations above 61 *μ*M did not inhibit platelet aggregation further. Interestingly, eugenol and elemicin exhibited dose-dependent inhibition of ADP (2 *μ*M)-induced platelet 5-HT release, although eugenol at a much lower concentration than elemicin (see Table 1 and Figure 4 (eugenol data only)). Concentration ranges of eugenol, elemicin and aspirin chosen to examine their inhibitory activity on human platelet 5-HT release were 2.8 × 10^−6^ to 2.8 × 10^−4^ M, 1.7 × 10^−4^ to 3.5 × 10^−3^ and 2.5 × 10^−6^ to 2.5 × 10^−4^ M, respectively. Despite structural similarities, there were remarkable differences in the level of activity between eugenol and elemicin [1]. Eugenol was up to 50 times more potent than elemicin, with IC_50_ values calculated to be 46.6 *μ*M and 1729.8 *μ*M, respectively. ASA is an irreversible inhibitor of platelet cyclooxygenase (COX) activity, and still remains the standard to which other anti-platelet drugs are often compared [28]. The dose-dependent effect of eugenol compared favorably to ASA (Figure 4), which exhibited an IC_50_ value of 46.1 *μ*M in these studies.

## 4. Discussion

Bioassay-directed fractionation of the DCM extract of *C. ambiguus* afforded the isolation and structural characterization of four compounds, eugenol, elemicin, eugenol methylether and *trans-*iso-elemicin. Like 1,8-cineole (the major active constituent of *Melaleuca*, *Eucalyptus* and *Prostanthera* sp.), eugenol and elemicin are volatile monoterpenoids (i.e., C_10_ compounds) with anti-inflammatory affects [29]. Indeed, other similar compounds have previously been isolated from *Cymbopogon* sp. including, limonene and *α*-terpineol, which are generally common components of citrus oils [30, 31]. Eugenol and elemicin have also been isolated from several other plants including *Myristica fragrans* (nutmeg) and *Syzygium aromaticum* (clove oil), where they are believed to be some of the major constituents responsible for the biological activities of these plants [29, 32, 33]. Elemicin has been identified previously in another Australian native, *Cymbopogon procerus* [3]; however, this is the first report of eugenol in the *Cymbopogon* sp. and indeed the first evidence for the presence of these compounds in the Australian native lemongrass species, *C. ambiguus*.

In these studies, eugenol was found to be up to 50-fold more active than elemicin at inhibiting 5-HT release from human platelets. From dose-dependant responses IC_50_ values of 46.6 *μ*M and 1729.8 *μ*M were determined, respectively, in our 5-HT release inhibition assay (Figure 4 and Table 1]. In previous studies using rabbit platelets, eugenol was found to be up to 1000 times more potent than elemicin and was found to compare favorably with indomethacin [34]. An additional study found eugenol to be 29 times more potent than aspirin at inhibiting arachidonic acid-induced human platelet aggregation [35]. Based on reports in the literature and the structural characteristics of eugenol methylether and *trans*-iso-elemicin, which were also isolated in the active fraction, these two compounds were not investigated further in our pharmacological studies [34]. Although, interestingly it has been demonstrated that eugenol methylether has anti-nociceptive effects on formalin-induced hyperalgesia [36]. However, our interests and discussion here focus on eugenol due to its principal activity in our bioassays.

**Figure 5:**
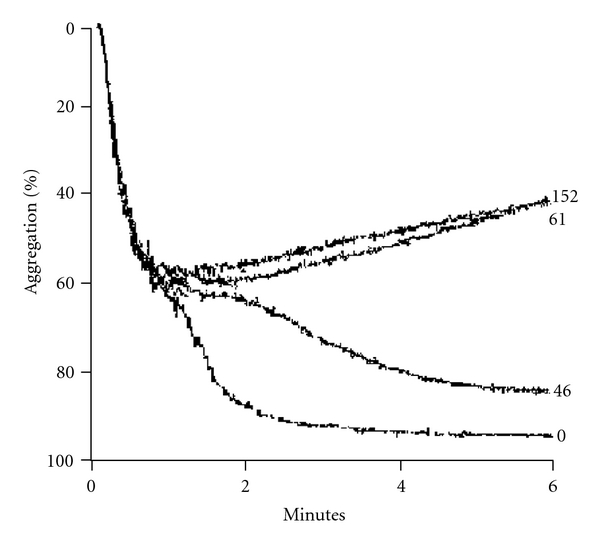
Platelet aggregation trace showing an example of the effect of low to high doses (0, 46, 61 and 152 *μ*M) of eugenol on ADP-induced platelet aggregation. The change in light transmission is represented as percentage of platelet aggregation using a four-channel platelet aggregometer. Results are duplicates obtained from PRP from two different individuals.

Studies have demonstrated that eugenol has anti-oxidant, anti-inflammatory, anti-platelet (using rabbit platelets), anti-nociceptive and anti-ulcerogenic effects [37–42]. Furthermore, this drug has also been shown to potentiate GABA_A_ transmission [43, 44]. Eugenol is reported to cause inhibition of thromboxane A_2_ and B_2_ formation, without any influence on the lipoxygenase pathway and appears to be an inhibitor of COX activity similarly to aspirin. In addition, more recent data indicates that this drug inhibits the rise in intracellular Ca^2+^ caused by collagen, adrenalin, ADP and AA [38]. Hence, eugenol appears to have pharmaceutical features that might be considered ideal characteristics of a migraine treatment. In support of these earlier studies [29], our results provide evidence that eugenol does not have significant effects on the primary phase of ADP induced platelet aggregation. Concentrations of eugenol above 61 *μ*M did not inhibit platelet aggregation further (Figure 5) and this result strongly suggests that eugenol selectively targets the second phase of platelet aggregation. This second phase of aggregation involves the “release reaction," which is dependent on cytosolic increases in Ca^2+^ and the activation of PLC (phospolipase C) and the COX pathway [45], which mediate platelet dense granule secretion. Therefore, 5-HT release could be completely inhibited even when aggregation is not. In line with this, high concentrations of eugenol that inhibited 5-HT release only inhibited the second phase of platelet aggregation (Figures 4 and 5]. The modulation of 5-HT release was the principal pathway we were interested in investigating.

Aspirin is a potent anti-inflammatory analgesic, known to cause inhibition of the COX pathway, namely the COX-1 and COX-2 isoforms of this enzyme. In general, aspirin is used to assist in prevention of heart attacks, strokes, arthritis, diabetes and migraines and may also slow the mental decline of old age or provide neuroprotection in a mouse model of Parkinson's disease [46, 47]. Previous studies have also shown that a single concentration of eugenol, inhibits both COX-1 and COX-2 enzymes [48]. Our results show that eugenol exhibits pharmacological effects that resemble those of aspirin and this data supports previous evidence that eugenol is a COX inhibitor and acts as a potent anti-platelet drug. Interestingly, eugenol is reported to exhibit analgesic properties [38] and has also been used to treat gastrointestinal upsets and chronic diarrhoea and is approved by the Food and Drug Administration of the USA. It is noteworthy that other traditional medicines known to modulate 5-HT activity have also been reported as treatments for gastrointestinal tract [49] and allergenic disorders [50]. Eugenol is also likely to have potential in the treatment of other diseases related to platelet aggregation, such as, thrombosis, transient ischemia, inflammation, tumor growth and promotion of atherosclerosis [51, 52]. However, there is only limited data relating to its potential use as an anti-headache/migraine or anti-platelet drug [29]. Few side effects have been reported in relation to the use of eugenol. One report on its use as a traditional dental material does however document local irritative and cytotoxic effects along with hypersensitivity reactions when used in contact with soft oral tissues [53].

## 5. Conclusions

**Figure 6:**
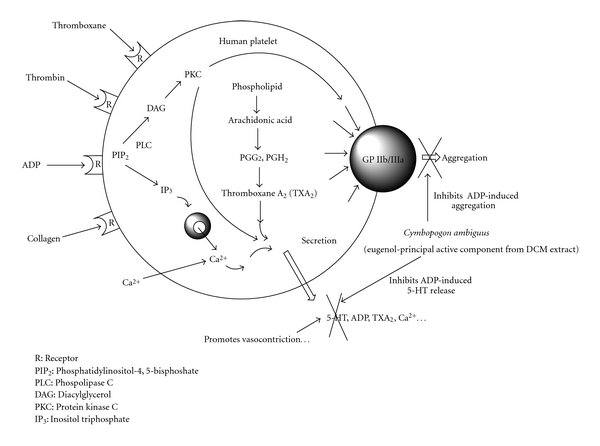
Schematic representation of platelet activation and the inhibitory effects of eugenol (from the DCM extract of *C. ambiguus*) on ADP-induced 5-HT release and platelet aggregation.

In conclusion, four known compounds (eugenol, elemicin, eugenol methyl ether and *trans*-isoelemicin) were isolated from the DCM extract of *C. ambiguus*. Eugenol and elemicin were identified as contributing the principal activity of this extract and demonstrated dose-dependent inhibition of ADP-induced human platelet [^14^C]5-HT release, with IC_50_ values of 46.6 *μ*M and 1729.8 *μ*M, respectively. Eugenol was also identified as having potent affects on the second-phase of platelet activation. The results of these studies provide evidence identifying specific constituents (principally eugenol) that have anti-platelet activity (Figure 6) that establish the basis of the therapeutic activity and traditional use of *C. ambiguus* as a non-conventional remedy for headache conditions.

## Figures and Tables

**Table 1:** IC_50_ values for components of *C. ambiguus* and aspirin against ADP-induced platelet 5-HT release.

Compound	IC_50_ (*μ*M)
Eugenol	46.6 ± 3.7
Elemicin	1729.8 ± 147.5
Acetylsalicylic acid (ASA, Aspirin)	46.1 ± 3.1

Results represent IC_50_ ± SEM (*n* = 4) as determined by the software of Graphpad prism (3.0).

## References

[1] Latz PK (1995). *Bushfires and Bushtucker. Aboriginal Plant Use in Central Australia*.

[2] Lassak EV, McCarthy T (1983). *Australian Medicinal Plants*.

[3] Barr A, Chapman J, Smith N, Beveridge M (1988). *Traditional Bush Medicines. An Aboriginal Pharmacopoeia. Aboriginal Communities of the Northern Territory of Australia*.

[4] Semple SJ, Reynolds GD, O’Leary MC, Flower RLP (1998). Screening of Australian medicinal plants for antiviral activity. *Journal of Ethnopharmacology*.

[5] Palombo EA, Semple SJ (2001). Antibacterial activity of traditional Australian medicinal plants. *Journal of Ethnopharmacology*.

[6] Rogers KL, Grice ID, Griffiths LR (2001). Modulation of *in vitro* platelet 5-HT release by species of *Erythrina* and *Cymbopogon*. *Life Sciences*.

[7] D’Andrea G, Welch KMA, Riddle JM, Grunfeld S, Joseph R (1989). Platelet serotonin metabolism and ultrastructure in migraine. *Archives of Neurology*.

[8] D’Andrea G, Cananzi AR, Perini F (1994). Decreased collagen-induced platelet aggregation and increased platelet arginine levels in migraine: a possible link with the NO pathway. *Cephalalgia*.

[9] Evers S, Quibeldey F, Grotemeyer K-H, Suhr B, Husstedt I-W (1999). Dynamic changes of cognitive habituation and serotonin metabolism during the migraine interval. *Cephalalgia*.

[10] Joseph R, Welch KMA, Grunfeld S, Oster SB, D’Andrea G (1988). Cytosolic ionized calcium homeostasis in platelets: an abnormal sensitivity to PAF-activation in migraine. *Headache*.

[11] Lingjaerde O, Monstad P (1986). The uptake, storage, and efflux of serotonin in platelets from migraine patients. *Cephalalgia*.

[12] Pukhal’skaya TG (1993). Effects of steroid hormones and anti-migraine drugs on serotonin transport in platelets of patients suffering from migraine and in those of healthy subjects. *Bulletin of Experimental Biology and Medicine*.

[13] Tozzi-Ciancarelli MG, De Matteis G, Di Massimo C, Marini C, Ciancarelli I, Carolei A (1997). Oxidative stress and platelet responsiveness in migraine. *Cephalalgia*.

[14] D’Andrea G, Granella F, Leone M, Perini F, Farruggio A, Bussone G (2006). Abnormal platelet trace amine profiles in migraine with and without aura. *Cephalalgia*.

[15] Ferrari MD (1998). Migraine. *Lancet*.

[16] Fioroni L, D’Andrea G, Alecci M, Cananzi A, Facchinetti F (1996). Platelet serotonin pathway in menstrual migraine. *Cephalalgia*.

[17] Hering R, Glover V, Pattichis K, Catarci T, Steiner TJ (1993). 5HT in migraine patients with medication-induced headache. *Cephalalgia*.

[18] Panconesi A (2008). Serotonin and migraine: a reconsideration of the central theory. *Journal of Headache and Pain*.

[19] Hamel E (2007). Serotonin and migraine: biology and clinical implications. *Cephalalgia*.

[20] Schwedt TJ (2007). Serotonin and migraine: the latest developments. *Cephalalgia*.

[21] Jagroop IA, Mikhailidis DP (2000). An investigation of the serotonergic effects of fenfluramine, dexfenfluramine and dexnorfenfluramine using platelets as neuronal models. *Platelets*.

[22] Joseph R, Welch KM, D’Andrea G (1989). Serotonergic hypofunction in migraine: a synthesis of evidence based on platelet dense body dysfunction. *Cephalalgia*.

[23] SDBS (2001). *Integrated Spectral Database System for Organic Compounds*.

[24] Giesbrecht AM, Franca NC, Gottlieb OR, Da Rocha AI (1974). The neolignans of Licaria canella. *Phytochemistry*.

[25] Enqiques RG, Chavez MA (1980). Propenylbenzenes from *Guatteria gaumeri*. *Phytochemistry*.

[26] Rogers KL, Grice ID, Griffiths LR (2000). Inhibition of platelet aggregation and 5-HT release by extracts of Australian plants used traditionally as headache treatments. *European Journal of Pharmaceutical Sciences*.

[27] Groenewegen WA, Heptinstall S (1990). A comparison of the effects of an extract of feverfew and parthenolide, a component of feverfew, on human platelet activity in-vitro. *Journal of Pharmacy and Pharmacology*.

[28] Bennett JS (2001). Novel platelet inhibitors. *Annual Review of Medicine*.

[29] Janssens J, Laekeman GM, Pieters LAC, Totte J, Herman AG, Vlietinck AJ (1990). Nutmeg oil: identification and quantitation of its most active constituents as inhibitors of platelet aggregation. *Journal of Ethnopharmacology*.

[30] De Oliveira AC, Ribeiro-Pinto LF, Paumgartten JR (1997). In-vitro inhibition of CYP2B1 mono oxygenase by beta-myrcene and other monoterpenoid compounds. *Toxicology Letters*.

[31] Dudai N, Weinberg ZG, Larkov O, Ravid U, Ashbell G, Putievsky E (2001). Changes in essential oil during enzyme-assisted ensiling of lemongrass (*Cymbopogon citrates* Stapf.) and lemon eucalyptus (*Eucalyptus citiodora* Hook). *Journal of Agricultural and Food Chemistry*.

[32] Srivastava KC (1993). Antiplatelet principles from a food spice clove (Syzgium aromaticum L). *Prostaglandins Leukotrienes and Essential Fatty Acids*.

[33] Srivastava KC, Malhotra N (1991). Acetyl eugenol, a component of oil of cloves (*Syzygium aromaticum* L.) inhibits aggregation and alters arachidonic acid metabolism in human blood platelets. *Prostaglandins Leukotrienes and Essential Fatty Acids*.

[34] Rasheed A, Laekeman GM, Vlietinck AJ, Janssens J, Hatfield G, Totte J (1984). Pharmacological influence of nutmeg and nutmeg constituents on rabbit platelet function. *Planta Medica*.

[35] Raghavendra RH, Naidu KA (2009). Spice active principles as the inhibitors of human platelet aggregation and thromboxane biosynthesis. *Prostaglandins Leukotrienes and Essential Fatty Acids*.

[36] Yano S, Suzuki Y, Yuzurihara M (2006). Antinociceptive effect of methyleugenol on formalin-induced hyperalgesia in mice. *European Journal of Pharmacology*.

[37] Sharma JN, Srivastava KC, Gan EK (1994). Suppressive effects of eugenol and ginger oil on arthritic rats. *Pharmacology*.

[38] Chen S-J, Wang M-H, Chen I-J (1996). Antiplatelet and calcium inhibitory properties of eugenol and sodium eugenol acetate. *General Pharmacology*.

[39] Chen Y-C, Chen J-J, Chang Y-L (2004). A new aristolactam alkaloid and anti-platelet aggregation constituents from Piper taiwanense. *Planta Medica*.

[40] Ohkubo T, Shibata M (1997). The selective capsaicin antagonist capsazepine abolishes the antinociceptive action of eugenol and guaiacol. *Journal of Dental Research*.

[41] Capasso R, Pinto L, Vuotto ML, Di Carlo G (2000). Preventive effect of eugenol on PAF and ethanol-induced gastric mucosal damage. *Fitoterapia*.

[42] Lee K-G, Mitchell A, Shibamoto T (2000). Antioxidative activities of aroma extracts isolated from natural plants. *BioFactors*.

[43] Aoshima H, Hamamoto K (1999). Potentiation of GABAA receptors expressed in Xenopus oocytes by perfume and phytoncid. *Bioscience, Biotechnology, and Biochemistry*.

[44] Szabadics J, Erdelyi L (2000). Pre- and postsynaptic effects of eugenol and related compounds on *Helix pomatia* L. neurons. *Acta Biologica Hungarica*.

[45] Puri RN, Colman RW (1997). ADP-induced platelet activation. *Critical Reviews in Biochemistry and Molecular Biology*.

[46] Bovill JG (1997). Mechanisms of actions of opioids and non-steroidal anti-inflammatory drugs. *European Journal of Anaesthesiology*.

[47] Teismann P, Ferger B (2001). Inhibition of the cyclooxygenase isoenzymes COX-1 and COX-2 provide neuroprotection in the MPTP-mouse model of Parkinson’s disease. *Synapse*.

[48] Everts B, Wahrborg P, Hedner T (2000). COX-2-specific inhibitors—the emergence of a new class of analgesic and anti-inflammatory drugs. *Clinical Rheumatology*.

[49] Tominaga K, Kido T, Ochi M, Sadakane C, Mase A, Okazaki H (2009). The traditional Japanese medicine Rikkunshito promotes gastric emptying via the antagonistic action of the 5-HT_3_ receptor pathway in rats. *Evidence-Based Complementary and Alternative Medicine*.

[50] Kumar A, Prasad R, Jogge NM, Bhojraj S, Emerson SF, Prabakar S (2008). Herbex-kid inhibits immediate hypersensitivity reactions in mice and rats. *Evidence-Based Complementary and Alternative Medicine*.

[51] Cecil RP, Susan H, Eleftherios PD, George S, David MG (1995). The red wine phenolics trans-resveratrol and quercetin block human platelet aggregation and eicosanoid synthesis:implications of protection against coronary heart disease. *Clinica Chimica Acta*.

[52] Ruggeri ZM (2002). Platelets in atherothrombosis. *Nature Medicine*.

[53] Sarrami N, Pemberton MN, Thornhill MH, Theaker ED (2002). Adverse reactions associated with the use of eugenol in dentistry. *British Dental Journal*.

